# Supplementary material for: Genome-wide meta-analysis of alcohol use disorder in East Asians
Source: Neuropsychopharmacology. 2022 Jan 29;47(10):1791–7. doi: 10.1038/s41386-022-01265-w (PMC9372033; doi:10.1038/s41386-022-01265-w)

Supplementary Figure 1. Forest plot of the top SNPs. a. Converted ORs of rs1229984*C allele in each cohort and effect size by meta-analyses. b. Converted ORs of rs3782886*C allele in each cohort and effect size by meta-analyses. N is sample size, Neffective is the effective sample size used in the meta-analysis, AF is allele frequency. [a]The OR was converted from effect size of LMM. [b]The IVW meta-analysis using the converted log(OR) as input. [c]The original meta-analysis using effective sample size-weighted method, this is not for comparison but for visual inspection.

			a. rs1229984*C allele		b. rs3782886*C allele	
**Cohorts**	**N**	**$N_{effective}$**	**AF**	**p-value**	**AF**	**p-value**
MVP-EAA[a]	6,955	2,521	0.33	0.04	0.14	$1.00x10^{-11}$
Thai METH-MEGA[a]	2,370	2,112	0.57	$1.03x10^{-8}$	0.08	$2.76x10^{-8}$
Thai METH-GSA[a]	532	387	NA	NA	0.10	0.07
Han Chinese-GSA	3,381	1,796	0.34	$1.03x10^{-12}$	0.17	$1.96x10^{-8}$
Han Chinese-Cyto[a]	313	271	0.32	0.18	0.21	$1.50x10^{-6}$
IVW meta-analysis[b]	13,551	7,087	0.41	$3.68x10^{-30}$	0.13	$2.03x10^{-42}$
Meta-analysis[c]	13,551	7,087	0.41	$3.35x10^{-17}$	0.13	$1.68x10^{-29}$

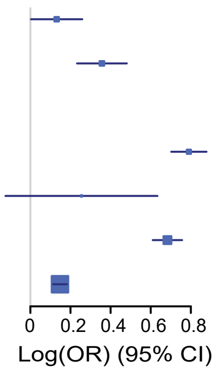

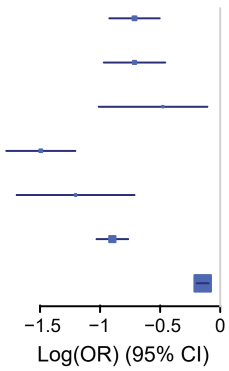

Supplement: Supplementary file 1 — Supplementary Figure 1 [file 41386_2022_1265_MOESM1_ESM.pdf]
